# Characterization of alcohol‐related seizures in withdrawal syndrome

**DOI:** 10.1002/epi4.12906

**Published:** 2024-01-27

**Authors:** Bettina Kata Kádár, Janka Gajdics, Ildikó Katalin Pribék, Bálint Andó, Bence András Lázár

**Affiliations:** ^1^ Addiction Research Group, Department of Psychiatry, Albert Szent‐Györgyi Medical School University of Szeged Szeged Hungary

**Keywords:** alcohol withdrawal syndrome, alcohol‐related seizure, delirium tremens, kindling, severity of alcohol withdrawal syndrome

## Abstract

**Objective:**

Alcohol‐related seizures (ARS) are one of the most important consequences of alcohol withdrawal syndrome (AWS). However, demographic and clinical characteristics, and furthermore, the relationship of ARS with delirium tremens (DT), have not yet been evaluated in detail. Therefore, the aim of the present study was to reveal the correlates of ARS and examine the interaction of ARS with the occurrence of DT and with the severity of AWS.

**Methods:**

In the retrospective study (Study 1) 2851 medical charts of inpatient admissions characterized by AWS and DT were listed. Demographic and clinical variables of ARS were assessed. In the follow‐up study (Study 2), patients admitted with AWS without (*N* = 28) and with (*N* = 18) ARS were enrolled. Study 1 was performed between 2008 and 2023, and Study 2 was performed in 2019 in Hungary. To determine the severity of AWS, the Clinical Institute Withdrawal Assessment Scale for Alcohol, Revised (CIWA‐Ar) was used. ARS is a provoked, occasional seizure; therefore, patients with epilepsy syndrome were excluded from the two studies. Statistical analyses were performed by the means of chi‐square tests, multinomial logistic regressions, mixed ANOVA, and derivation.

**Results:**

The occurrence of DT, the history of ARS, and somatic co‐morbidities were found to be risk factors for the appearance of ARS. ARS was proved to be a risk factor for the development of DT. In the follow‐up study, there was no difference in the decrease of CIWA‐Ar scores between the groups.

**Significance:**

Our present findings support the likelihood of kindling, which is one of the most important mechanisms underlying the development of ARS, but do not directly prove its presence. Additionally, our results revealed that the severity of AWS is not influenced by the presence of ARS.

**Plain Language Summary:**

Provoked, occasional seizures during AWS are defined as ARS. In the present study, predictors and interactions of these seizures with DT—the most severe form of withdrawal—and with the severity of withdrawal were examined in retrospective and follow‐up studies. The present study shows that a history of withdrawal seizures, the occurrence of DT, and somatic comorbidities are predictors of the development of seizures. Furthermore, our findings suggest that the presence of seizures does not influence the severity of withdrawal.

**Key Point**

1. Occurrence of seizures during alcohol withdrawal syndrome is about 10%.
2. History of delirium tremens (DT) and seizures are predictors of seizures.
3. Co‐morbid somatic disorders are predictors of developing seizures.
4. Alcohol‐related seizures are a predictor of developing DT.
5. The severity of withdrawal syndrome may not be dependent on the presence of seizures.

## 1 INTRODUCTION

Alcohol withdrawal syndrome (AWS) is one of the most important consequences and outcomes of alcohol use disorder (AUD) and occurs after a period of relative or absolute reduction of alcohol intake.^1^, ^2^, ^3^ It has been revealed that about 50% of patients diagnosed with AUD develop AWS. Although AWS comprises a broad spectrum of symptoms with various severity, it has been demonstrated that about 20% of withdrawal syndromes have complications such as alcohol‐related seizures (ARS) and delirium tremens (DT), which entail an elevated incidence of mortality.^1^, ^2^, ^3^, ^4^, ^5^, ^6^

The term ARS is mainly reserved for generalized tonic–clonic seizures (“rum fits”) that occur during AWS, and their characteristics were first described by Victor and Brausch.^7^ According to the definition and concept by the International League Against Epilepsy^8^, ^9^ and articles about AWS,^4^, ^5^ the seizures occurring during an AWS are provoked seizures. Therefore, in the present work, the definition of ARS as a provoked seizure was used. Although several reports have suggested that ARS is closely related to severe AWS (SAWS),^7^ usually all seizures that are associated with alcohol use, including AUD and DT, are defined as ARS.

Most studies have indicated that the prevalence of ARS among alcohol‐dependent individuals is about 5%–30%.^1^, ^2^, ^3^, ^4^, ^5^, ^10^, ^11^ Reports have also revealed a relatively high rate (1%–3%) of mortality due to the development of status epilepticus, sudden unexpected death in epilepsy, or traumatic brain injury.^1^, ^2^, ^3^, ^12^, ^13^ In addition, considering that ARS may be a predictor of the development of DT, it may have importance in reducing the lethal consequences of the delirium syndrome (e.g., arrhythmias) to prevent seizures during the withdrawal state.^1^, ^3^, ^6^, ^14^ Previous reports also indicate that alcohol consumption, AUD severity, and dual disorder frequency changed during the COVID‐19 pandemic, and these changes may lead to the development of new diagnostic and therapeutic approaches.^15^, ^16^, ^17^ Furthermore, the therapeutic guidelines on SAWS are contradictory regarding benzodiazepine (BZD) and non‐BZD medications such as antiepileptic medications.^1^, ^2^, ^5^, ^18^ Therefore, the examination of SAWS, especially withdrawal seizures has a significant importance. However, there is no detailed data about the distribution pattern of ARS in AWS and DT.

It has been suggested that ARS occurs during the first period of AWS, which is 12–48 h after the cessation of alcohol.^3^, ^5^ ARS is usually described as a generalized tonic–clonic motor seizure that generally presents once during withdrawal.^9^ Some authors have indicated that the presence and the history of ARS are strong risk factors for developing DT, which are associated with severe medical complications and increased inpatient morbidity.^4^, ^7^, ^19^, ^20^ However, the relationship between the presence and history of ARS and DT has not yet been evaluated in detail.

Nevertheless, earlier animal and human studies have suggested that the history of moderate or severe withdrawal syndromes increases the risk of developing AWS and complicated forms (e.g., seizures, DT) of withdrawal.^21^, ^22^ Over the past few decades, this kindling hypothesis has been supported by various reports.^23^, ^24^, ^25^, ^26^, ^27^ Moreover, it has been evaluated that since repeated seizures may render the central nervous system more excitable, a history of ARS can lead to the development of an epileptogenic state of the brain, which may be a potent risk factor for developing a future seizure during withdrawal syndrome.^28^, ^29^ Various reports have also revealed that the genetic and molecular background of withdrawal seizures is different from that of other provoked seizures.^30^, ^31^, ^32^, ^33^, ^34^, ^35^

Additionally, it has been suggested that the severity of AWS may not be dependent on the presence of ARS.^10^ Furthermore, it has been indicated that delayed climax of the severity of withdrawal, higher prevalence of cortical lesions, and history of DT and detoxification admissions could be determining factors to predict the presence of ARS.^10^, ^36^

The etiology of ARS and DT is complex and involves various distinct demographic and clinical characteristics. Furthermore, the clinical characteristics of ARS are still poorly understood. Additionally, the interaction between seizures and delirious symptoms has not yet been clearly revealed; hence, detecting the risk factors of ARS is of critical importance regarding the high mortality rate of complicated forms of AWS.

Based on the above summarized data, the aims of the present study were to evaluate the demographic and clinical characteristics of ARS, to evaluate the risk factors for developing seizures, to reveal the interplay of ARS with DT, and to examine the relationship between the severity of AWS and the presence of ARS.

## 2 MATERIAL AND METHODS

### 2.1 Study 1

#### 2.1.1 Setting

The aim of the study was to review the medical charts of inpatient admissions with the principal diagnosis of mental and behavioral disorders due to the use of alcohol according to the International Classification of Diseases, Tenth Revision (ICD‐10) in the Department of Psychiatry, University of Szeged, Hungary, between January 01, 2008 and January 01, 2023.

The inclusion criteria were for the medical charts to have the principal diagnoses of alcohol withdrawal state (AWS; F10.30) and/or alcohol withdrawal state with delirium (DT; F10.40). The ARS variable was defined as the occurrence of ICD‐10‐related diagnoses for occasional, provoked seizures. Medical charts with the diagnoses of epilepsy syndromes and BZD use disorder were excluded.

The patients hospitalized during this period were treated with the same pharmacological therapy (fixed‐schedule doses of BZD). Electroencephalograms (EEGs) were not recorded routinely during the treatment of AWS in our Department; therefore, these data are not available. Furthermore, other predictors, such as drinking history, were unknown due to the retrospective nature of the study.

The present results are part of a larger study to be published elsewhere.

Demographic variables (age and sex), somatic‐ and psychiatric co‐morbidities, levels of electrolytes (sodium and potassium), liver enzymes (gamma‐glutamyl transferase (GGT), serum glutamic‐oxaloacetic transaminase (SGOT), serum glutamic pyruvic transaminase (SGPT), and SGOT/SGPT quotient), and the history of AWS, DT, and ARS were collected from medical charts of inpatient admissions.

Two groups were formed based on the occurrence of ARS: AWS coursed with (ARS^+^) and without ARS (ARS^−^). Demographic variables, laboratory parameters, and the co‐occurrence of co‐morbid disorders were analyzed in the two groups. The evaluation of risk factors for the development of ARS was determined. ARS as a risk factor for DT was assessed as well.

This study was performed in line with the principles of the Declaration of Helsinki. The study was approved by the Human Investigation Review Board of the University of Szeged (ethical approval numbers: 30/2016‐SZTE; 82/2022‐SZTE).

#### 2.1.2 Statistical analysis

All statistical analyses were performed using IBM SPSS 24.^37^

Chi square tests and independent sample *t*‐tests were used to compare the ratio of the presence of ARS in the total sample and the ratio of demographic variables, laboratory parameters, and the ratio of co‐morbid disorders in the ARS^+^ and ARS^−^ subgroups and the DT^+^ and DT^−^ subgroups. Multinomial logistic regression models were used to determine the variables explaining the appearance of ARS and DT. The dependent variables were the occurrence of ARS and the appearance of DT in the case of ARS. The independent variables were those that showed a significant difference between the two groups, and when DT was the dependent variable, ARS was the independent variable.

### 2.2 Study 2

#### 2.2.1 Setting

With the aim of assessing the relationship between the presence of ARS and the severity of AWS, patients admitted with a diagnosis of alcohol withdrawal state (AWS; F.10.30) at the inpatient units of the Department of Psychiatry, University of Szeged, Hungary between January 01, 2019 and December 31, 2019 were included in this study.

Inclusion criteria were as follows: (1) diagnosis of alcohol dependence syndrome (F.10.20.); (2) diagnosis of AWS (F.10.30.); (3) a minimum of seven points on the first CIWA‐Ar; and (4) fixed‐schedule regimen with chlordiazepoxide. Exclusion criteria were as follows: (1) symptoms of DT; (2) the presence of epilepsy syndrome; (3) clinically significant changes in electrolyte levels and liver enzymes; (4) clinically significant somatic‐ and/or neurological disorders; (5) diagnosis of epilepsy syndrome; and (6) BZD use disorder. The patients hospitalized during this period were treated with the same pharmacological therapy (fixed‐schedule doses of BZD).

Following informed consent, a test pack was administered a total of six times every 2 days for 10 days to each patient who voluntarily enrolled in the study. The study of alcohol consumption habits and withdrawal symptoms was part of the inpatient care. The tests were recorded by an experienced physician.

The present results are part of a larger study. This study was performed in line with the principles of the Declaration of Helsinki. The study was approved by the Human Investigation Review Board, University of Szeged (ethical approval number: 28/2018‐SZTE).

#### 2.2.2 Measurement methods

At first, a set of demographic questions and the Alcohol Use Disorders Identification Test (AUDIT) were taken in the form of an interview. During the next five visits, the Clinical Institute Withdrawal Assessment of Alcohol, Revised (CIWA‐Ar)^38^, ^39^ was administered. Furthermore, laboratory parameters (sodium levels, SGGT, and SGOT‐SGPT quotient) and the occurrence of ARS were recorded.

#### 2.2.3 Statistical analysis

All statistical analyses were performed using IBM SPSS 24.^37^

An independent sample *t*‐test was used to compare the mean of the AUDIT scores between the ARS^+^ and ARS groups. A mixed ANOVA was used to evaluate the changes in the CIWA‐Ar scores.

## 3 RESULTS

### 3.1 Study 1

#### 3.1.1 Sample characteristics

In the analyses, 2851 medical charts of inpatient (*N* = 1630) admissions were included. The sample consisted of 19.6% female and 80.4% male patient admissions. The mean age was 50.44 years (SD = 11.311). AWS without DT was the diagnosis of 85.3% (*N* = 2431) of appearances, and 14.7% (*N* = 420) were with DT. The occurrence of ARS in the total sample was 9.7% (*N* = 276). The percentage of the complicated form of AWS was 22.2% (*N* = 634) and the percentage of the diagnosis of ARS and DT together in the total sample was 2.2% (*N* = 62).

The mean age in the ARS^+^ group was 49.21 years (SD = 11.264), while it was 50.57 years (SD = 11.311) in the ARS‐ group. The difference between the two groups had a tendency level significance (*t*[2849] = 1.902, *p* = 0.057). The percentage of female appearances was 14.9% in the ARS^+^ group and 20.1% in the ARS^−^ group, and this difference was significant (χ^2^ = 4.319, *p* = 0.038, OR = 0.694). There was also a significant difference between the two groups in the prevalence of DT (χ^2^ = 14.544, *p* < 0.001, OR = 1.794; Table 1).

**TABLE 1:** Characteristics of the ARS^+^ and ARS^−^ subgroups.

	ARS^−^ (*n* = 2575)	ARS^+^ (*n* = 276)
Age (SD)	50.57 (11.311)	49.21 (11.264)
Female	20.1%	14.9%*
Male	79.9%	85.1%*
Prevalence of DT	13.9%	22.5%*
Somatic co‐morbidities	53.5%	63.8%*
Psychiatric co‐morbidities	45.8%	40.9%
Potassium (mean, SD)	4.04 (0.562)	4.91 (8.994)
GGT (mean, SD)	278.070 (471.253)	308.899 (402.883)
SGOT (mean, SD)	71.18 (77.556)	78.9 (91.679)
SGPT (mean, SD)	55.79 (95.515)	57.37 (56.947)
SGOT/SGPT quotient (mean, SD)	1.467 (0.818)	1.501 (0.865)

Abbreviations: ARS, alcohol related seizure; DT, delirium tremens; GGT, gamma‐glutamyl transferase; SGOT, serum glutamic‐oxaloacetic transaminase; SGPT, serum glutamic pyruvic transaminase; SD, standard deviation.

* *p* < 0.05.

#### 3.1.2 Characteristics of the ARS ^+^ and ARS ^−^ subgroups

The co‐occurrence of somatic and psychiatric co‐morbidities, history of AWS, DT, and ARS, and levels of electrolytes and liver enzymes (GGT, SGOT, SGPT, and SGOT/SGPT quotient) were analyzed in the ARS^+^ and ARS^−^ subgroups. The percentage of co‐existing somatic disorders was significantly higher in the ARS^+^ group (63.8%) than in the ARS^−^ group (53.5%) (χ^2^ = 10.569, *p* = 0.001, OR = 1.529). However, there was no significant difference in the occurrence of co‐existing psychiatric disorders between the ARS^+^ (40.9%) and ARS^−^ (45.8%) subgroups (χ^2^ = 2.398, *p* = 0.121, OR = 0.820).

There was a significant difference in the history of DT (*t*[299.899] = −3.544, *p* < 0.001) and the history of ARS (*t*[278.506] = −7.021, *p* < 0.001) between the ARS^+^ (*M*_hDT_ = 0.22; *M*_hARS_ = 1.08) and ARS^−^ (*M*_hDT_ = 0.09; *M*_hARS_ = 0.09) groups. Nevertheless, there was no significant difference between the two groups in the history of AWS without DT (*t*[2849] = −1681, *p* = 0.093).

Electrolyte levels were in a normal range in both groups. Elevated GGT, SGOT, SGPT levels, and SGOT/SGPT quotients were determined in the two groups. There was no significant difference in the laboratory parameters between both groups. Table 1 shows the characteristics of the ARS^+^ and ARS^−^ subgroups.

#### 3.1.3 Predictors of the development of ARS

A multinomial logistic regression model was created to determine the possible predictors of ARS. The model consisted of variables that showed a significant difference between the ARS^−^ and ARS^+^ groups. Thus, sex, DT, somatic co‐morbidity, history of ARS, and history of DT were included in the model, which showed a significant interconnection (χ^2^ = 186.066, *p* < 0.001) and had 90.7% certainty. The presence of DT, the history of ARS, and somatic co‐morbidity played a significant explanatory role in the development of ARS. Table 2 shows the results of the regression model.

**TABLE 2:** The multinomial logistic regression model of ARS.

ARS	*B*	SE	df	*p*	OR	95% confidence interval for OR	
						Lower bound	Upper bound
History of DT	0.05	0.056	1	0.371	1.051	0.942	1.172
History of ARS	0.679	0.073	1	<0.001	1.973	1.708	2.278
Male	0.365	0.19	1	0.055	1.441	0.992	2.093
Female	0		0				
DT^−^	−0.608	0.164	1	<0.001	0.544	0.394	0.751
DT^+^	0		0				
Somatic comorbidity^−^	−0.375	0.143	1	0.009	0.687	0.52	0.909
Somatic comorbidity^+^	0		0				

Abbreviations: ARS, alcohol related seizure; *B*, regression coefficient; df, degrees of freedom; DT, delirium tremens; OR, odds ratio; *p*, significance; SE, standard error.

#### 3.1.4 ARS as a predictor for developing DT

A multinomial regression model was performed to examine ARS as a predictor of DT. The model was significant (χ^2^ = 21.404, *p* < 0.001) and had 90.3% certainty. According to the model, ARS significantly increases the probability of DT. Table 3 shows the results of the regression model.

**TABLE 3:** The multinomial logistic regression model of DT.

	*B*	SE	df	*p*	OR	95% confidence interval for OR	
						Lower bound	Upper bound
ARS^−^	−0.74	0.15	1	<0.001	0.477	0.356	0.641
ARS^+^	0		0				

Abbreviations: ARS, alcohol related seizure; *B*, regression coefficient; df, degrees of freedom; DT, delirium tremens; OR, odds ratio; *p*, significance; SE, standard error.

### 3.2 Study 2

#### 3.2.1 Sample characteristics

The sample consisted of 15 female (32.6%) and 31 male (67.4%) patients (*N* = 46). The mean age was 44.261 years (SD = 8.928). Two subgroups (ARS^+^, ARS^−^) were created based on the presence of seizures during the course of AWS. Patients with ARS had slightly higher scores (*M* = 30.389, SD = 6.463) on the AUDIT scale compared to the ARS^−^ group (*M* = 30.143, SD = 5.024); however, the difference was not significant (*t* (44) = −0.145, *p* = 0.886). Table 4 shows the sample characteristics.

**TABLE 4:** Characteristics of the ARS^−^ and ARS^+^ subgroups in the follow‐up study.

	ARS^−^ (*n* = 28; 60.9%)	ARS^+^ (*n* = 18; 39.1%)
Age (SD)	44.929 (8.886)	43.222 (9.149)
Female	28.6%	38.9%
Male	71.4%	61.1%
AUDIT score (SD)	30.143 (5.024)	30.389 (6.463)

Abbreviations: ARS, alcohol related seizure; AUDIT, Alcohol Use Disorder Identification Test; SD, standard deviation.

#### 3.2.2 Changes in the CIWA‐Ar scores

Mixed ANOVA and derivation were used to evaluate the difference in the changes in CIWA‐Ar scores between the ARS^+^ and ARS^−^ groups. In the case of the main effect of CIWA‐Ar scores, the sphericity is not satisfied (χ^2^[14] = 216.390, *p* < 0.001); therefore, the degrees of freedom of the ANOVA were corrected using the Greenhouse–Geisser method (ε = 0.347). The CIWA‐Ar scores were significantly decreasing during the six visits (*F*[1.736, 76.397] = 193.989, MSE = 5819.722, *p* < 0.001). The Bonferroni post hoc test showed that the scores of all visits differed significantly from each other, except for the 5th and 6th visits' scores (*p* = 0.130). There was no significant difference in the decrease of CIWA‐Ar scores between the subgroups (*F*(1, 44) = 16.784, MSE = 388, *p* = 0.536).

**FIGURE 1:**
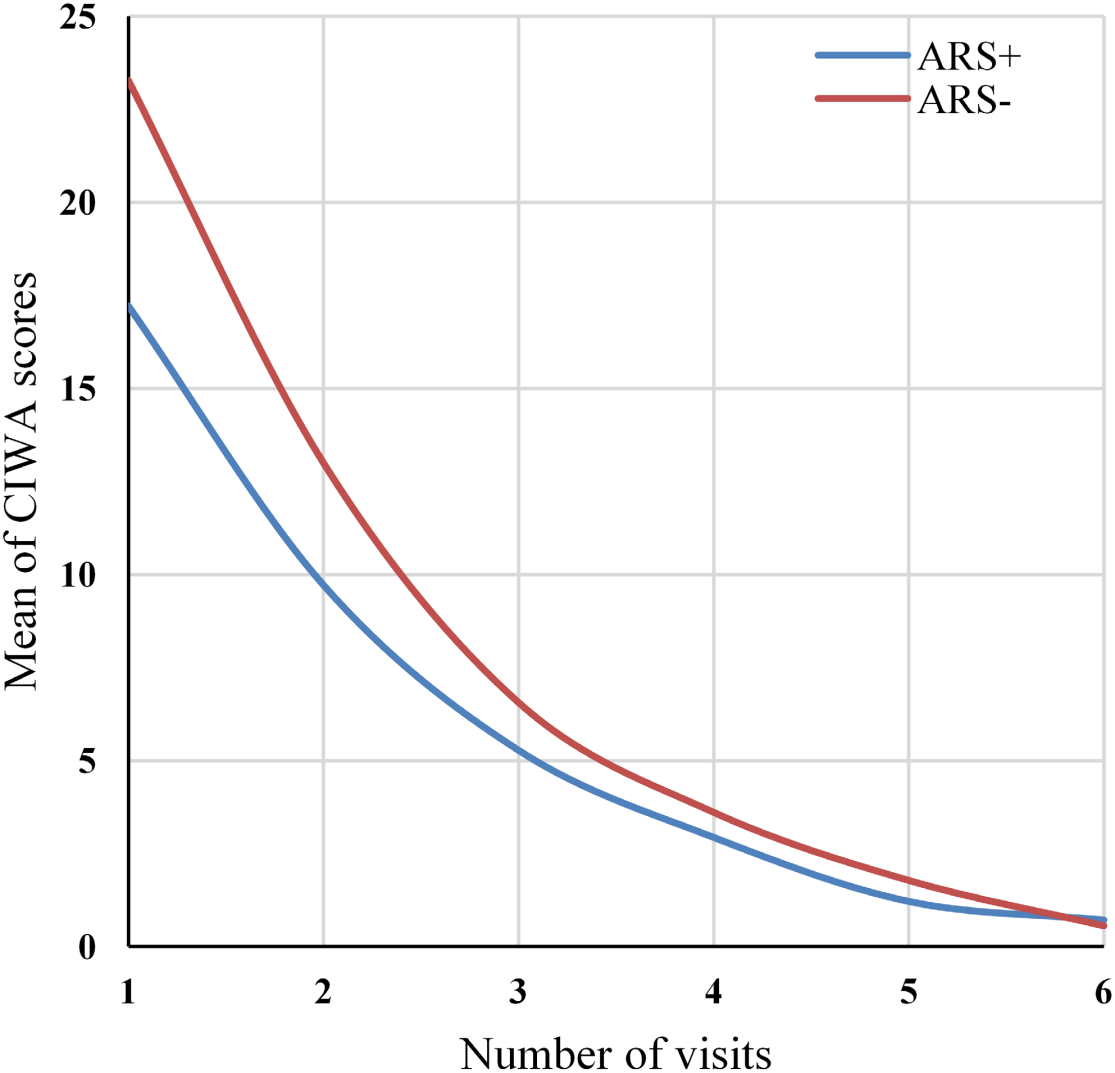
Decrease of CIWA‐Ar scores in the ARS^−^ and ARS^+^ subgroups. ARS, alcohol related seizure; CIWA, Clinical Institute Withdrawal Assessment of Alcohol, Revised.

To compare the characteristics of the decrease in CIWA‐Ar scores between the ARS^+^ and ARS^−^ subgroups, an index number was created for every patient's six visits. A quadratic curve was fitted to the six points (CIWA‐Ar scores). The slope of the curve at the given six points was calculated, and the six obtained values were averaged. The difference between the ARS^+^ and ARS^−^ groups regarding these CIWA‐Ar index numbers was calculated with an independent sample *t*‐test. There was no significant difference between both groups (*t* (44) = −1.143, *p* = 0.515), therefore the decrease of the CIWA‐Ar scores during the six visits was not dissimilar (Figure 1).

## 4 DISCUSSION

Previous studies have demonstrated that the prevalence of complicated withdrawal syndromes, including ARS and DT, is between 5% and 20% among hospitalized patients, and its presence strongly increases morbidity and mortality.^4^, ^5^ Hence, evaluating the risk factors for the development of ARS has critical importance in the prevention of the lethal consequences of AWS. Although several studies have suggested that patients with ARS may comprise a specific subgroup of alcohol‐dependent individuals,^24^, ^30^, ^31^, ^32^, ^33^, ^34^, ^35^ little is known about its prevalence, demographic variables, clinical characteristics, predictors, and interplays with AWS and DT. So, in the present comprehensive study, the demographic and clinical characteristics, as well as predictors of ARS, were assessed using two samples. The interplay of ARS with the development of DT and with the severity of AWS was also examined.

Our present data support and extend previous observations^2^, ^3^ by showing that about 20% of inpatient admissions are presented with a complicated form of AWS, and the percentage of ARS was about 10% in the total sample. However, in the literature, there is no data about the concomitance of ARS and DT in AWS. Our results showed in a clinical sample that the percentage of the co‐occurrence of ARS and DT was 2.2%.

In the present study, differences between the admissions based on the occurrence of ARS and the predictors for developing ARS during the withdrawal syndrome were also determined. Our results show that the majority of admissions with the diagnosis of seizure were male patients, and the presence of DT, the co‐occurrence of somatic co‐morbidities, and the history of DT and ARS were significantly higher among appearances where ARS occurred. Furthermore, the presence of DT, the co‐occurrence of somatic co‐morbidities, and the history of ARS were predictors of developing seizures. Additionally, in agreement with Eyer and his colleagues, our present findings suggest that laboratory parameters were not risk factors for the occurrence of ARS.^10^

Overall, according to the meta‐analyses of Wood et al.,^36^ most of the clinical studies examined specifically the risk factors for the development of DT and the complicated forms of AWS; however, the risk factors for ARS are still poorly understood. Our present study supports the key role of the kindling hypothesis and the co‐occurrence of other somatic disorders by revealing the explanatory role of the history of ARS and DT and the presence of somatic co‐morbidities in the eventuality of withdrawal seizures.^3^, ^10^, ^40^, ^41^

Kim et al.^42^ have demonstrated that one‐third of patients with a history and presence of ARS developed DT during withdrawal syndrome. Moreover, some reports have suggested that individuals diagnosed both with ARS and DT may be representing a genetically homogenous subgroup.^24^, ^30^, ^31^, ^32^, ^33^, ^34^, ^35^ By demonstrating that the presence of DT has an explanatory role in developing ARS, our results support these observations.

Moreover, although several reports have evaluated that various factors such as older age, male sex, high blood level of homocysteine, low platelet count, low potassium level, and psychiatric and somatic co‐morbidities can be risk factors for developing DT,^19^, ^36^, ^43^, ^44^, ^45^ the interplay of the occurrence of ARS and DT has not been evaluated in detail. Some studies have suggested that the ARS in medical history can increase the risk of the development of DT via the kindling mechanism.^36^ Additionally, in the present study, our results revealed that the occurrence of seizures during withdrawal increases the risk for the eventuality of DT. One previous report indicated that quantitative EEG might be a helpful tool for detecting patients with a high risk of developing DT after a withdrawal seizure during the course of AWS.^46^

In our follow‐up study, the severity of alcohol dependence was measured by using the AUDIT, and the present results revealed that although significant differences between the two groups were not detected, patients with ARS showed slightly higher AUDIT scores. Previous observations have also demonstrated that there are no significant differences in the AUDIT scores between both groups.^44^ Previously, AUDIT has been examined as a predictor tool for the development of AWS.^47^, ^48^ These observations revealed that AUDIT alone is not suitable to detect the risk of AWS.

Recent studies have indicated that there is a strong relationship between the occurrence of ARS and the severity of AWS.^4^, ^5^ However, it has been reported that there is no difference in the maximal score of the Alcohol Withdrawal Scale when comparing the severity of AWS among patients with or without ARS.^10^ Nonetheless, it has also been revealed that the severity of AWS shows a delay. Our findings support and extend these observations by showing that there are no significant differences between the two groups regarding the admission score, the maximal score, and the decrease in severity. Furthermore, a meta‐analysis by Woo and his colleagues revealed that there is no causal relationship between alcohol consumption and the development of seizures.^49^ In the present study, CIWA‐Ar was used, which is a widely recommended tool^38^, ^39^ for the diagnosis and monitoring of withdrawal symptoms. During the past few years, some authors have suggested that CIWA‐Ar has various limitations in its application for screening the severity of withdrawal syndrome.^4^, ^5^ These results can explain our observations; however, it could also be hypothesized that, regarding the specified features of ARS and DT, the occurrence of ARS may be independent from the severity of withdrawal.

### 4.1 Limitations

The present study has several limitations. Firstly, the generalizability of our results is limited since the data collection was conducted at one regional hospital in Hungary. Furthermore, the major limitation of our retrospective study is that medical charts were examined; hence, our results only reflect clinical data associated with the course of AWS.

Several previous reports have examined the risk factors of complicated AWS, and the majority of our findings support their observations. The present paper assessed the risk factors of ARS in a retrospective examination of data from a 15‐year span, and the relationship between the severity of withdrawal syndrome and the occurrence of seizures was analyzed in a follow‐up study. Moreover, other clinically relevant information, such as drinking history and EEG records, was unknown due to the retrospective nature of the study.

Additionally, in our follow‐up study, the severity of AWS was measured with one tool and without vital signs. Moreover, a small number of patients were examined during a relatively short 1‐year period. In the future, a more detailed assessment of the severity of AWS will be needed.

Overall, further studies are necessary to determine the interaction between the occurrence of ARS and the severity of withdrawal. However, our results may be important for better understanding the interplay between the occurrence of ARS, the development of DT, and the severity of AWS.

## 5 CLINICAL RELEVANCE

In conclusion, the present study evaluated the potential risk factors for the occurrence of ARS and demonstrated the importance of ARS in the eventuality of DT. Preventing the severe and potentially lethal consequences of AWS is extremely important for clinicians; therefore, identification of risk factors for complicated forms of withdrawal syndrome can lead to prompt treatment of these conditions. For instance, the use of antiepileptic medications in the subgroup of individuals with the risk factors of ARS can prevent the development of ARS and may reduce the risk of DT.^2^, ^5^ Moreover, our results may help us better understand the interplay of these specific states of AWS. Additionally, in agreement with previous observations, our present work suggests that ARS and DT may comprise a special subgroup of withdrawal syndrome that can be independent of the severity of AWS.

## AUTHOR CONTRIBUTION

BAL, BA, IKP, JG, and BKK designed and conceptualized the study. BAL, JG, and BKK conducted a literature search. BAL, BA, IKP, and JG contributed to data collection and organization. BAL, BA, IKP, and JG conducted the data analysis. All authors contributed to the data interpretation, and BKK, BAL, and JG wrote the first version of the manuscript. All authors contributed to providing edits and feedback to manuscript drafts and contributed and approved the final version of the manuscript.

## FUNDING INFORMATION

This work was supported by the Hetényi Géza Grant (SZTE‐ÁOK‐KKA‐2019‐HG). Janka Gajdics and Bence András Lázár were supported by the ÚNKP‐22‐3‐SZTE‐251 and ÚNKP‐22‐4‐SZTE‐306 New National Excellence Program of the Ministry for Culture and Innovation from the source of the National Research, Development, and Innovation Fund.

## CONFLICT OF INTEREST STATEMENT

The authors have no conflict of interest to declare.

## ETHICS STATEMENT

This study was performed in line with the principles of the Declaration of Helsinki. The study was approved by the Human Investigation Review Board, University of Szeged (ethical approval numbers: 30/2016‐SZTE; 28/2018‐SZTE; 82/2022‐SZTE).

## PATIENT CONSENT STATEMENT

Due to the retrospective nature of Study 1, informed consent of the patients was not required because the study analyzed anonymous clinical data of the patients. In Study 2, written informed consent was obtained from each participant.

## EPILEPSIA OPEN ETHICAL PUBLICATION STATEMENT

We confirm that we have read the journal's position on issues involved in ethical publication and affirm that this report is consistent with those guidelines.

## Data Availability

The dataset of the study is available from the corresponding author (Bettina Kata Kádár) upon request. This anonymized dataset has been generated from the registered official health insurance patient flow of the university clinic, and due to the official data protection policy, these data are preferred not to be made fully available but only on request. Further enquiries can be directed to the corresponding author (Bettina Kata Kádár).
